# Epidural analgesia combined with transversus abdominis plane block with liposomal bupivacaine reduces pain after caesarean delivery: a double-blind, randomised controlled trial

**DOI:** 10.3389/fmed.2025.1653815

**Published:** 2025-08-29

**Authors:** Wei Ma, Yanxi Long, Shanshan Ye, Qiang Tao, Yang Wang, Tao Xu

**Affiliations:** ^1^Department of Anaesthesiology, International Peace Maternity and Child Health Hospital, School of Medicine, Shanghai Jiao Tong University, Shanghai, China; ^2^Shanghai Key Laboratory of Embryo Original Diseases, Shanghai, China

**Keywords:** epidural analgesia, transversus abdominis plane, liposomal bupivacaine, caesarean delivery, postoperative pain

## Abstract

**Background:**

Sustained release of bupivacaine can be achieved by encapsulating bupivacaine within multivesicular liposomes, providing localised analgesia for up to 72 h. This study aimed to evaluate whether a multimodal analgesic approach integrating epidural analgesia with liposomal bupivacaine enhanced transversus abdominis plane block could extend the interval to initial opioid use and thus reduce the total post-operative opioid requirements in women undergoing caesarean section.

**Methods:**

Women scheduled for elective caesarean delivery under combined spinal-epidural anaesthesia were randomly assigned to a liposomal bupivacaine or placebo group. All participants were given 0.75% ropivacaine 15 mg intrathecally at L3–L4 interspace. Before surgical closure, 0.6 mg of epidural hydromorphone was administered. Bilateral ultrasound-guided lateral transversus abdominis plane blocks were performed after surgery. The liposomal bupivacaine group received 133 mg liposomal bupivacaine in 20 mL fluid per side, whereas the placebo control group received 20 mL saline per side. Postoperative analgesia included scheduled oral acetaminophen and self-administered boluses of oxycodone as needed. The primary outcomes were 24- and 48-h oxycodone consumption.

**Results:**

A total of 128 women were enrolled. The median [interquartile range (IQR)] postoperative cumulative oxycodone consumption was significantly lower in the liposomal bupivacaine group than in the placebo group at 24 h [2 (0–5) mg vs. 4 (1–8) mg, *p* = 0.009] and 48 h [8 (0–13) mg vs. 10 (4–18) mg, *p* = 0.022], and the median (IQR) interval to first patient-controlled analgesia use was significantly longer in the liposomal bupivacaine group than in the placebo group [22 (12–48) vs. 8 (4–18) h; *p* < 0.001].

**Conclusion:**

Epidural hydromorphone combined with liposomal bupivacaine enhances transversus abdominis plane block, prolongs analgesia duration and reduces opioid requirements in the first 48 h after caesarean-section.

**Clinical trial registration:**

https://www.chictr.org.cn/showproj.html?proj=237618, Identifier [ChiCTR2400087477].

## 1 Introduction

Postoperative pain following caesarean-section delivery is a concern. Poorly managed acute pain can progress to chronic post-caesarean-section pain (CPCSP) (1, 2), which has a severe negative effect on quality of life, including maternal psychological well-being and infant care (3, 4). Uncontrolled acute pain is associated with a 2.5-fold increased risk of chronic pain and postnatal depression (4, 5). Ensuring optimal postoperative analgesia in women undergoing caesarean section is essential to prevent CPCSP and improve maternal outcomes.

Since the first trial investigating transversus abdominis plane (TAP) block for caesarean delivery was published in 2008 (6), several randomised controlled trials have demonstrated that the addition of TAP blocks has significant analgesic and opioid-sparing effects (7–9). However, subsequent trials revealed that the efficacy of TAP blocks is inferior to that of 100–200 μg of intrathecal morphine (10–12). This may be because of the shorter duration of analgesic effect of ropivacaine and bupivacaine, which are used in TAP blocks, compared with that of intrathecal morphine.

Liposomal bupivacaine (LB), a sustained-release formulation designed to provide up to 120 h of localised analgesia (13), provides a promising alternative to opioid analgesics. Encapsulating bupivacaine within multivesicular liposomes achieves gradual drug release, potentially bridging the analgesic gap between single-injection peripheral nerve blocks and extended-duration neuraxial opioids (14).

In China, where preservative-free morphine is not available, epidural opioids are widely used for postoperative analgesia. We hypothesised that the incorporation of LB-enhanced TAP block into a multimodal analgesic regimen anchored by epidural hydromorphone would significantly prolong the duration of effective analgesia and thereby reduce postoperative opioid requirements following caesarean delivery. We conducted a clinical trial to investigate this hypothesis.

## 2 Methods

### 2.1 Study design and participants

This double-blind, randomised controlled trial was approved by the Institutional Scientific Research and Clinical Trials Committee of International Peace Maternity and Child Health Hospital (GKLW-A-024-032-01) and was registered in advance with the Chinese Clinical Trials Registry (Registration No.: ChiCTR2400087477).

Pregnant women scheduled for elective caesarean delivery under spinal-epidural anaesthesia were enrolled between 1 August 2024 and 15 April 2025. Eligible patients were women aged 20–40 years, classified as American Society of Anaesthesiologists (ASA) physical status II, carrying singleton pregnancies at 37–42 weeks of gestation, who requested postoperative analgesia. The exclusion criteria included contraindications to neuraxial anaesthesia, allergy to study medications (LB, ropivacaine, hydromorphone, oxycodone, or acetaminophen), body mass index (BMI) > 36 kg/m^2^, gastrointestinal disorders, a history of substance abuse, or refusal to participate. Participants were withdrawn if protocol deviations occurred (e.g., epidural catheter failure, or noncompliance with oral analgesia).

### 2.2 Randomisation and blinding

Randomisation was performed using SPSS-generated random numbers (1:1 ratio), with assignments sealed in opaque envelopes. Envelopes were distributed to participants during preoperative assessments and opened by anaesthetists postoperatively to prepare study medications. Owing to visible differences between LB and saline, the anaesthetists administering the TAP block were unblinded, while the participants, research assistant in charge of follow-up, and data analysts remained blinded to the group allocation.

### 2.3 Trial procedure

Participants were recruited by a research assistant on the day before the surgery, informed of study protocols, and provided written informed consent. The Chinese version of the 15-item Quality of Recovery (QoR-15) scale was used to assess the participant’s preoperative condition. Each participant was then provided with a sealed randomisation envelope. Participants were instructed to fast from midnight prior to surgery.

On arrival in the operating room, intravenous access was established, and standard monitoring including electrocardiography, non-invasive blood pressure measurement, and pulse oximetry was initiated. Combined spinal-epidural anaesthesia was administered to the L3–L4 interspace and 15 mg of 0.75% ropivacaine was injected intrathecally, followed by epidural catheter placement. Sensory block level was assessed using pin-prick to ensure that the block reached to or above T6 before surgery; otherwise a 5-mL dose of 1% lidocaine was administered through the epidural catheter.

During the surgery, obstetricians administer oxytocin, carbetocin, ergometrine, or prostaglandin-based uterotonic agents based on the patient’s uterine contractions, with oxytocin being the standard intraoperative choice. The number of uterotonic agents used is documented before the patient leaves the operating room.

Before surgical closure, 0.6 mg of epidural hydromorphone (diluted in 6 mL saline) was administered. Participants were provided with a patient-controlled intravenous analgesia (PCIA) device (ZZB-III, APON Corp, Nantong, Jiangsu, China) containing oxycodone 30 mg in 60 mL saline, provided as 2-mL PCIA boluses with a 10-min lockout interval and no background infusion, after the surgery.

Immediately after completing the surgery, the randomisation envelope was opened by the anaesthetist, and bilateral ultrasound-guided lateral TAP blocks were performed. The LB group received 133 mg LB in 20 mL fluid per side, whereas the placebo group received 20 mL saline per side. Postoperative analgesia included scheduled oral acetaminophen (500 mg every 6 h for 48 h). Participants were permitted to self-administer PCIA boluses when the numeric rating scale (NRS) score of their abdominal pain, measured on a scale of 0–10, was >3.

A research assistant documented the 24- and 48-h oxycodone consumption, and interval to the first PCIA, and assessed resting pain, bed mobility pain, uterine palpation pain, and ambulation pain using the NRS at 6, 12, 24, and 48 h postoperatively; collected QoR-15 scores at 24 and 48 h; and recorded analgesia-related adverse effects. The analgesic pump was discontinued 48-h postoperatively, and the analgesic satisfaction score was assessed using a 5-point Likert scale.

### 2.4 Primary and secondary outcomes

The primary outcomes were oxycodone consumption within the first 24- and 48 h postoperatively. The secondary outcomes included interval to first PCIA of oxycodone; resting and bed mobility NRS scores at 6, 12, 24, and 48 h postoperatively; worst NRS scores in postoperative 12 h during uterine palpation; 24- and 48-h ambulation NRS pain scores; side effects of analgesia; 24- and 48-h QoR-15 scores; and analgesia satisfaction 48 h after surgery.

### 2.5 Sample size calculation and statistical analysis

The required sample size was calculated using PASS 2022. Due to the lack of prior data on oxycodone consumption amongst patients receiving epidural hydromorphone in conjunction with intravenous oxycodone following caesarean sections, sample size estimation was based on our clinical experience using the incidence of moderate-to-severe pain (NRS > 3) during ambulation at 24 h, which was the trigger for oxycodone consumption in our trial. In our clinic, the incidence of moderate-to-severe pain (NRS > 3) was about 60% amongst parturients during ambulation at 24 h. Assuming a 30% absolute reduction (from 60% to 30%), the required sample size was calculated as 53 patients per group (*α* = 0.05, power = 90%). Allowing for a 20% attrition rate, the target sample size was set at 128 participants, with 64 participants per group.

The “survminer” R software package was used to generate Kaplan–Meier curves and the log-rank test was used to compare the probability of participants having PCIA between the LB and placebo groups. Violin Plots of NRS for resting and bed mobility at 6, 12, 24, and 48 h postoperatively were generated for each group using the “ggplot2” R software package. The differences and effect sizes of outcomes related to postoperative analgesia effect were calculated using the “effsize” R software package. These statistical analyses were performed using R 3.4.4 (R Foundation for Statistical Computing, Vienna, Austria). Statistical significance was defined as a two-tailed *p*-value < 0.05.

Continuous data were presented as the mean ± SD or median and interquartile range (IQR), and groups were compared using *t-*tests or Mann–Whitney U tests, as appropriate. Categorical variables were presented as frequencies and percentages and groups were compared using chi-square or Fisher’s exact tests. Cliff’s delta was used as a nonparametric measure of effect size of LB compared with placebo, with values close to 0 signifying no effect and values close to −1 or +1 signifying a greater effect. These statistical analyses were performed using SPSS for Windows, version 26.0 (IBM Corp, Armonk, NY, USA). Statistical significance was defined as a two-tailed *p-*value < 0.05.

## 3 Results

### 3.1 Participant flow and baseline characteristics

**Figure 1:**
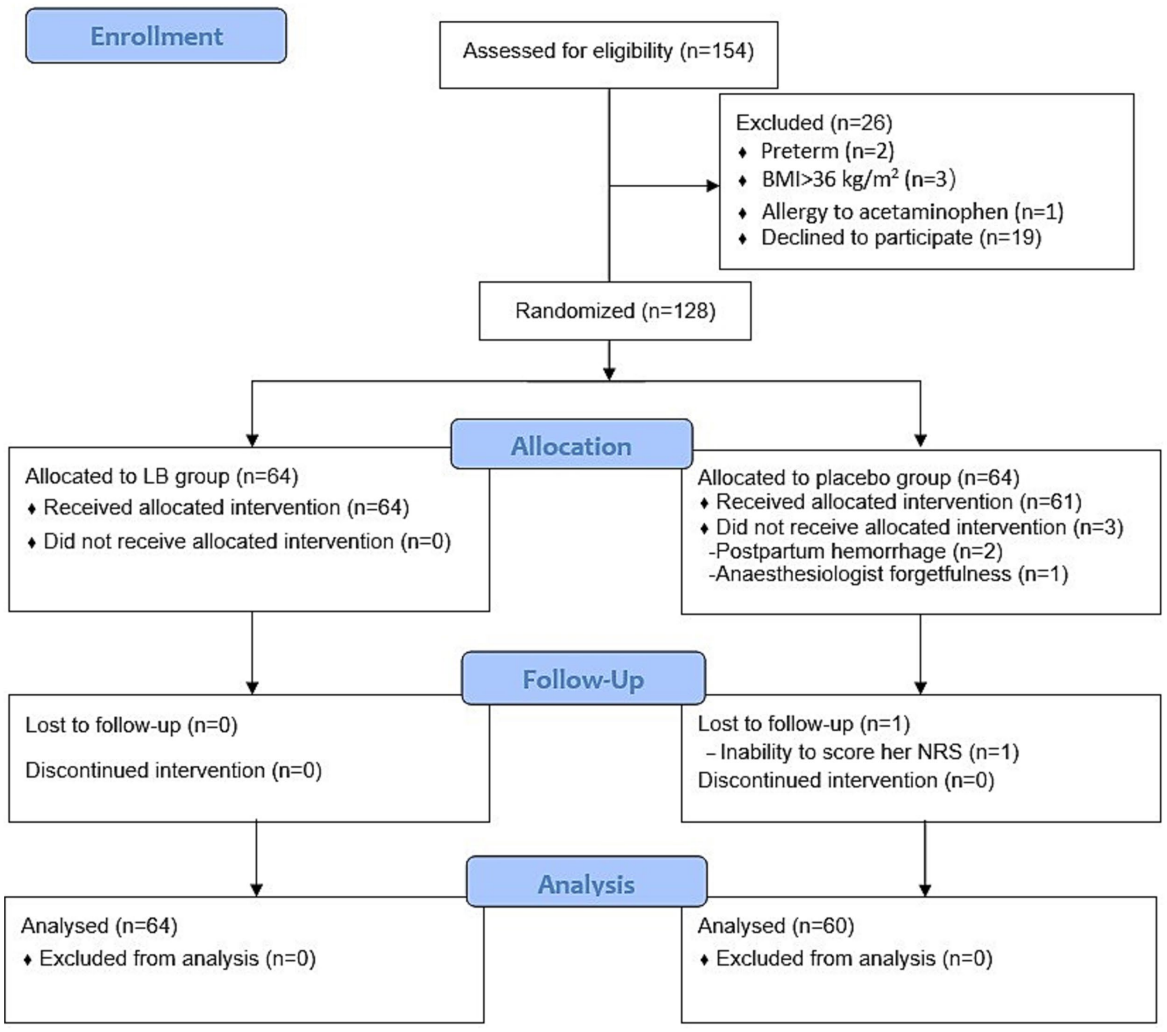
Flow diagram.

The CONSORT flow diagram (Figure 1) outlines participant enrolment and allocation. A total of 154 potential were identified, of whom 26 were excluded because of preterm delivery, BMI > 36 kg/m^2^, allergy to acetaminophen, or refusal to participate. After these exclusions, a total of 128 participants were enrolled in the study. Three participants in the placebo group did not receive TAP block after surgery (two cases with postpartum haemorrhage exceeding 1,000 mL did not undergo TAP block due to concerns of coagulopathy potentially causing local hematoma. One case was withdrawn due to researcher forgetfulness) and one participant in the placebo group was unable to score her pain degree. These four participants were excluded from the analysis, leaving 64 participants in the LB group and 60 participants in the placebo group.

The baseline demographic and clinical characteristics were comparable between the LB and placebo groups, with no significant differences in age, anthropometric measures, obstetric history, neonatal birth weight, or intraoperative parameters (all *p* > 0.05). The mean gestational age was slightly higher in the LB group than in the placebo group (39.0 ± 1.3 vs. 38.4 ± 1.7 weeks; *p* = 0.044) but this difference was not clinically significant. The types of uterotonic agents administered did not differ significantly between groups (Table 1).

**Table 1:** Parturient characteristics and baseline assessments.

	LB group (*n* = 64)	Placebo group (*n* = 60)	*p*-value
Age, year	33.5 ± 3.9	34.2 ± 4.2	0.295
Height, cm	162.7 ± 4.4	161.8 ± 4.0	0.203
Weight, kg	72.1 ± 9.3	71.0 ± 8.7	0.500
Gravidity, *n*	1 (1, 2)	1 (1, 2)	0.074
Parity, *n*	0 (0, 0)	0 (0, 0)	0.268
Gestational weeks, w	39.0 ± 1.3	38.4 ± 1.7	0.044
Baseline QoR-15 score	146 (143, 148)	145 (143, 147)	0.169
Baby birth weight, g	3,168 ± 351	3,194 ± 437	0.712
Numbers of uterotonic agents used during surgery, *n* (%)			
1	39 (60.9)	27 (45.0)	
2	18 (28.1)	28 (46.7)	
3	7 (10.9)	5 (8.3)	0.102
Surgery time, min	42.8 ± 7.4	45.0 ± 7.7	0.097
Blood loss, mL	205 ± 55	225 ± 88	0.114

Data are presented as mean ± standard deviation, median (interquartile range), or number (percentage) as appropriate.

QoR-15: 15 items of quality of recovery scale.

### 3.2 Primary outcome

The median (IQR) cumulative oxycodone consumption during the first 24 h after surgery was significantly lower in the LB group than in the placebo group [2 (0–5) mg vs. 4 (1–8) mg, *p* = 0.009]. The median (IQR) cumulative oxycodone consumption during the first 48 h after surgery was also significantly lower in the LB group than in the placebo group [8 (0–13) mg vs. 10 (4–18) mg, *p* = 0.022]. However, the median (IQR) oxycodone consumption during the period from 24 h to 48 h after surgery did not differ significantly between groups (Table 2).

**Table 2:** Outcomes related to post-operative analgesia effect.

	LB group (*n* = 64)	Placebo group (*n* = 60)	Median difference	*p*-value	Effect size with 95% CI
Primary outcomes					
Oxycodone consumption in 24 h, mg	2 (0, 5)	4 (1, 8)	−1 (−3–0)	0.009	−0.27 (−0.45 to −0.07)
Oxycodone consumption during 24–48 h, mg	4 (0, 8)	5 (1, 10)	−1 (−3–0)	0.253	−0.12 (−0.31–0.09)
Total oxycodone consumption in 48 h, mg	8 (0, 13)	10 (4, 18)	−3 (−6–0)	0.022	−0.24 (−0.42 to −0.03)
Secondary outcomes					
Interval to first PCA demand, hour	22 (12, 48)	8 (4, 18)	11.6 (6–17)	<0.001	0.49 (0.30–0.65)
Worst NRS pain score during uterine palpation in 12 h	2 (2, 3)	4 (3, 6)	−2 (−2 to −1)	<0.001	−0.50 (−0.65 to −0.31)
Ambulation NRS pain score in 24 h	4 (2.25, 5)	5 (4, 6)	−2 (−2 to −1)	<0.001	−0.45 (−0.61 to −0.26)
Ambulation NRS pain score in 48 h	2 (2, 3)	3.5 (3, 5)	−1 (−2 to −1)	<0.001	−0.40 (−0.57 to −0.20)
QoR-15 score at 24 h	138 (133, 143)	133 (122, 138)	5 (3–8)	0.002	0.38 (0.18–0.55)
QoR-15 score at 48 h	143 (138, 146)	137 (130, 142)	5 (3–8)	<0.001	0.42 (0.22–0.59)
5-Likert score of analgesic effect	5 (5, 5)	4 (4, 5)	0 (1–0)	<0.001	0.44 (0.27–0.58)

Data are presented as median (interquartile range).

NRS, numeric rating scale; PCA, patient-controlled analgesia; QoR-15, 15 items of quality of recovery scale; 95% CI, 95% confidence interval.

### 3.3 Secondary outcomes

**Figure 2:**
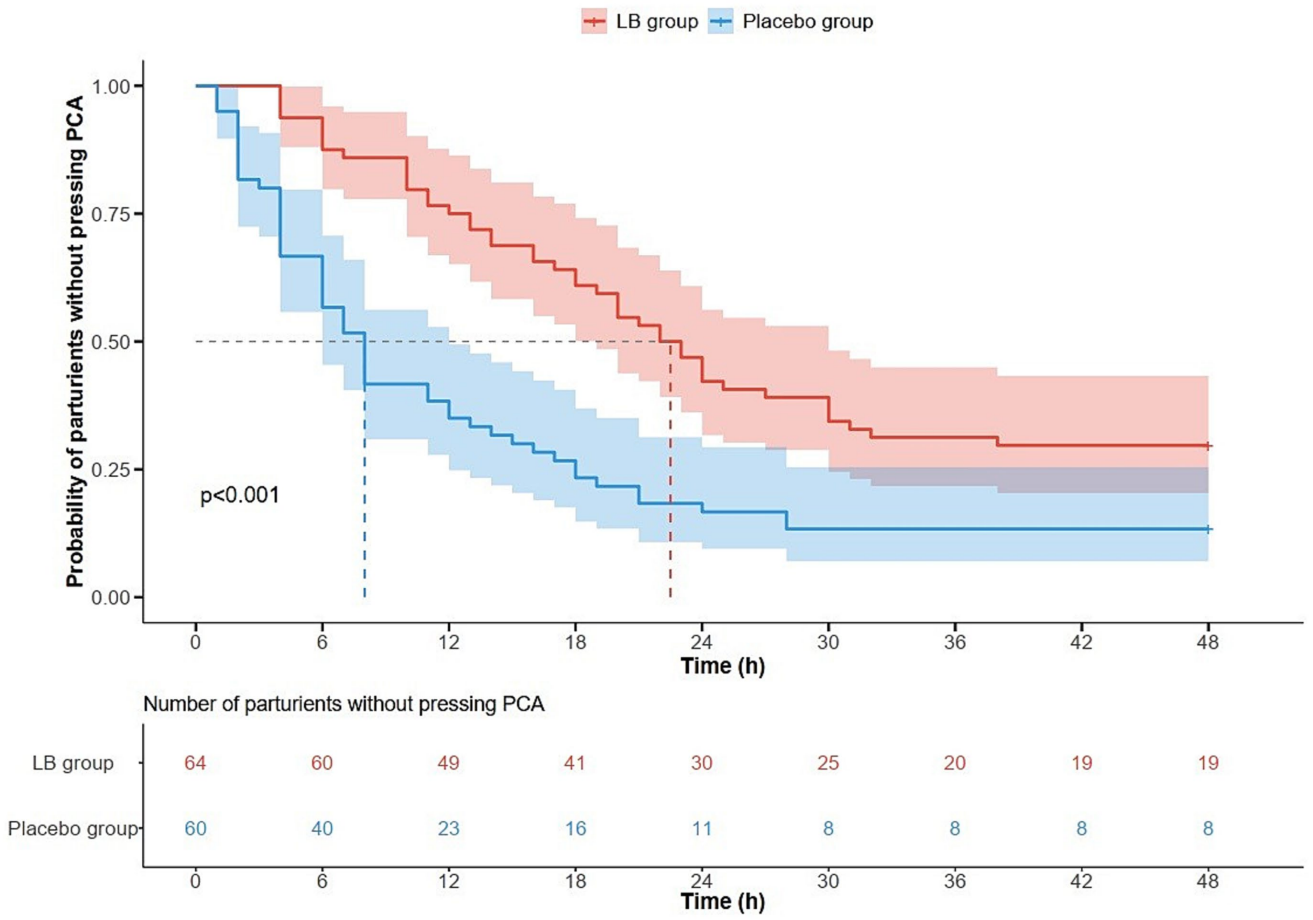
Kaplan–Meier curve of parturients without pressing PCA between LB and placebo group after caesarean delivery. The probability and numbers of parturients without pressing PCA at each postoperative time point are presented in this figure, and the time interval of the first oxycodone demand for 50% parturients was prolonged in the LB group [22 (12–48) vs. 8 (4–18) h, *p* < 0.001] compared with that of the placebo group. PCA, patient-controlled analgesia.

The Kaplan–Meier curves of the probability and numbers of participants without PCIA at each postoperative time-point are presented in Figure 2. The median (IQR) time to the first PCIA was significantly delayed in the LB group compared with the placebo group [22 (12–48) h vs. 8 (4–18) h, *p* < 0.001].

**Figure 3:**
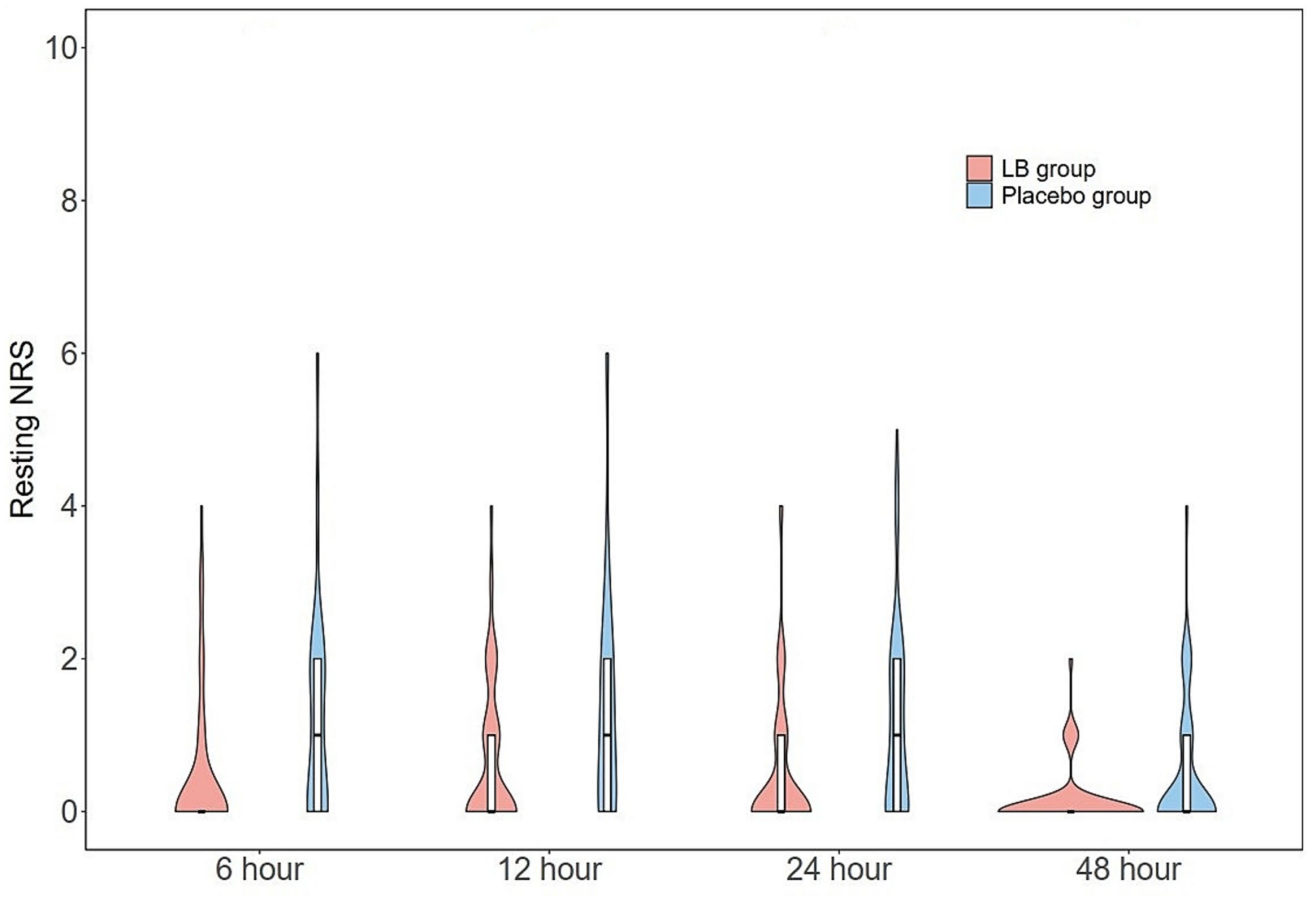
Violin plots of postoperative resting NRS scores at postoperative 6, 12, 24, and 48 h between LB and placebo group. The resting NRS scores in LB group were lower at postoperative 6 h (*p* < 0.001), at 12 and 24 h (*p* < 0.01), and at 48 h (*p* < 0.05) vs. placebo group. NRS, numeric rating scale.

**Figure 4:**
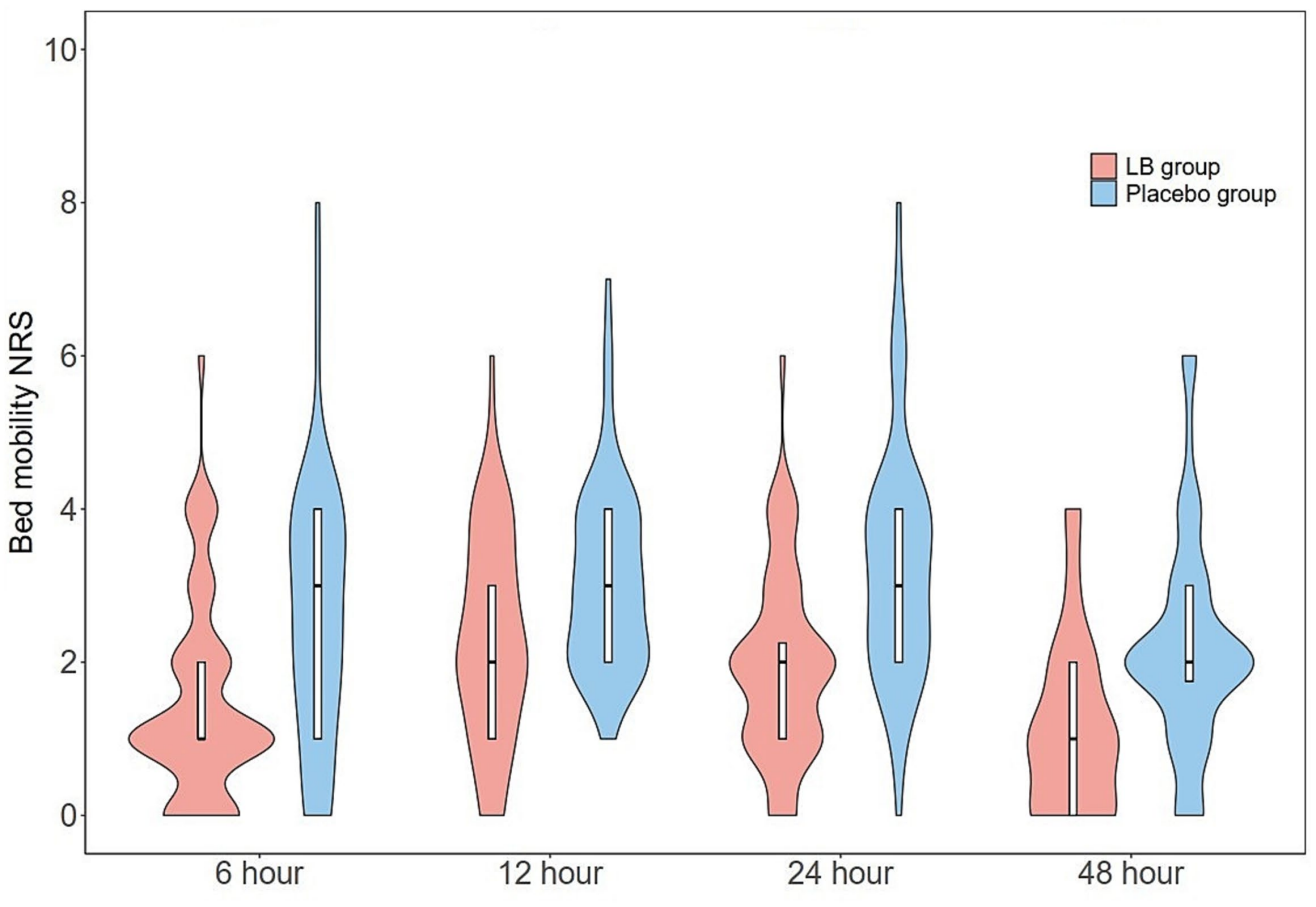
Violin plots of postoperative bed mobility NRS scores at postoperative 6, 12, 24, and 48 h between LB and placebo group. The bed mobility NRS scores in LB group were lower at postoperative 6 h (*p* < 0.0001), at 12 (*p* < 0.01), at 24 h and 48 h (*p* < 0.0001) vs. placebo group. NRS, numeric rating scale.

The resting and bed mobility NRS scores at 6, 12, 24, and 48 h postoperatively are presented in Figures 3, 4. The resting and bed mobility NRS scores in the LB group showed significantly better pain control than that in the placebo group at all time-points evaluated. The median (IQR) worst pain during uterine palpation at 12 h was significantly lower in the LB group than in the placebo group [NRS: 2 (2–3) vs. 4 (3–6); *p* < 0.001]. The ambulation pain scores at 24 h [4 (2.25–5) vs. 5 (4–6), *p* < 0.001] and 48 h [2 (2–3) vs. 3.5 (3–5), *p* < 0.001] postoperatively were also significantly lower in the LB group than in the placebo group (Table 2).

The QoR-15 scores revealed a 5-point greater median (IQR) recovery quality in the LB group compared with the placebo group at both 24 h [138 (133–143) vs. 133 (122–138), *p* = 0.002] and 48 h [143 (138–146) vs. 137 (130–142), *p* < 0.001] postoperatively. The median (IQR) analgesia satisfaction score at 48 h postoperatively was significantly higher in the LB group than in the placebo group [5 (5–5) vs. 4 (4–5), *p* < 0.001] (Table 2).

### 3.4 Safety

The adverse event profiles were comparable between the LB and placebo groups, with no statistically significant differences in the incidence of dizziness, nausea, vomiting, degree of pruritus, or urinary retention between the LB and placebo groups (Table 3).

**Table 3:** Side effects of post-operative analgesia.

	LB group (*n* = 64)	Placebo group (*n* = 60)	*p*-value
Dizziness, *n* (%)	5 (7.8%)	4 (6.7%)	0.806
Nausea, *n* (%)	4 (6.3%)	5 (8.3%)	0.655
Vomiting, *n* (%)	6 (9.4%)	7 (11.7%)	0.677
Pruritus, *n* (%)			
Mild	21 (32.8%)	23 (38.3%)	
Moderate	0	2 (3.3%)	
Severe	0	1 (1.7%)	0.260
Urine retention, *n* (%)	7 (10.9%)	4 (6.7%)	0.403

Data are presented as number (percentage).

## 4 Discussion

This study demonstrated that the multimodal analgesic approach integrating epidural analgesia with LB-enhanced TAP block reduces the cumulative total opioid requirements during the first 24 h and 48 h after caesarean delivery with lower NRSs in resting, bed mobility and ambulation, significantly extends the time delay to the initial opioid administration so that gain higher scores in QoR-15 and maternal satisfactory.

The TAP block was first described for caesarean delivery by McDonnell et al. (6) in 2008 and was later optimised using ultrasound guidance. TAP block has been shown to reduce somatic pain by targeting the thoracolumbar nerve branches (T6–L1) innervating the abdominal wall. Early studies highlighted their opioid-sparing potential in patients not receiving intrathecal morphine (ITM), and demonstrated significant reductions in postoperative pain scores and opioid consumption (7–9). However, the Enhanced Recovery After Caesarean Section (ERACS) guidelines prioritised neuraxial opioids such as ITM as the analgesic cornerstone, relegating TAP block to an adjunctive role (15). Despite showing promise in early studies, a recent review (16) and randomised controlled trials (10–12) have shown inconsistent opioid-sparing effects when TAP block is added to ITM-based protocols.

LB, encapsulating bupivacaine within multivesicular liposomes, achieves a sustained local analgesia effect for up to 120 h by gradual drug release (17, 18). The introduction LB reinvigorated interest of the use of TAP block in multimodal analgesia after caesarean delivery. Studies have demonstrated that patients treated with liposomal bupivacaine-enhanced TAP block shows reduced (13), or no more (19), morphine demand than those with ITM administration. Although TAP block can enhance postoperative multimodal analgesia, the magnitude of its analgesic effect varies depending on the type of local anaesthetic used (20).

Because preservative-free morphine is not available in China, we conducted a study to evaluate whether LB-enhanced TAP block augments epidural hydromorphone-based multimodal analgesia. This study demonstrated that the multimodal analgesic regimen integrating epidural analgesia with LB-enhanced lateral TAP block significantly prolonged the interval to first postoperative opioid requirement and resulted in reduced opioid consumption in 24 h and 48 h postoperatively. Furthermore, pain control in rest, bed mobility, ambulation, and uterine palpation in the 48 h postoperatively was significantly more effective in the LB group than in the placebo group. This study demonstrated that the time to first rescue analgesia in our method was comparable to that reported in recent literature for traditional intrathecal morphine (50 μg or 100 μg) combined with multimodal analgesia, with median of 23.5 h (95% CI: 15.2–28.3 h) and 22.9 h (95% CI: 13.8–28.3 h), respectively (21). Notably, this method showed a significant extension of the first rescue time compared to the use of liposomal bupivacaine (LB) as a single postoperative analgesic for caesarean delivery (median: 5 h, IQR: 3–15 h) (22). These further underscore the clinical advantages of our protocol in managing postoperative pain after caesarean delivery. However, when compared to the combination of intrathecal morphine with LB (median: 53.2 h, range: 2.3–345.2 h) (13), this advantage was less pronounced.

However, it should be noted that although the oxycodone consumption at 24 h and 48 h after liposomal bupivacaine-enhanced TAP blocks in this study showed statistically significant differences compared to the control group, the clinical relevance of these actual differences between the two groups may be limited. Additionally, no significant difference was observed in oxycodone usage between 24 h and 48 h between the two groups. This may be because during the early period before ambulation, while the control group had slightly higher pain NRS scores, neither group’s pain levels were sufficient to trigger patient-controlled analgesia. By the second day when ambulation was required, although the LB group demonstrated significantly lower pain scores during walking at 24 h, both groups frequently exceeded the threshold for PCIA activation, suggesting that standardized recovery protocols equalized opioid demand despite differences in nociceptive experiences between the LB and placebo groups. Furthermore, the lack of significant difference in opioid consumption between 24 h and 48 h postoperatively may reflect institutional protocols promoting early mobilization—a core principle of ERAS guidelines. This highlights the complex interplay between somatic pain control, behavioural recovery mandates, and opioid utilisation patterns in postoperative care (23). As the ERAS protocol may dilute the differences in opioid use between the two groups, future trials should control or stratify the amulation protocol to eliminate confounding factors introduced by ERAS.

In addition to epidural analgesia and TAP block, the multimodal analgesia regimen used in the study included scheduled oral acetaminophen and PCIA oxycodone. The epidural hydromorphone dose was 0.6 mg, which has been shown to provide superior postoperative analgesia than either higher or lower doses of epidural hydromorphone, while minimising the adverse effects (24, 25). Oral acetaminophen 500 mg was administered every 6 h, effectively controlling the total 24-h acetaminophen dose within the FDA-recommended maximum daily dose of 3,250 mg (26). The PCIA bolus dose was set at oxycodone 1 mg. Furthermore, this comprehensive multimodal analgesic strategy ensured the safety of postpartum analgesia following caesarean delivery and minimised interference with breastfeeding (27).

### 4.1 Strengths and limitations of the study

A strength of this study is the comprehensive evaluation of the analgesic effect of LB-enhanced TAP block using multiple endpoints: opioid rescue doses; interval to first PCIA demand; resting, bed mobility, ambulation and uterine palpation NRS pain scores; QoR-15; satisfaction score, and the use of Cliff’s delta to measure the effect size. These measures demonstrated the analgesic effect of LB-enhanced TAP block.

This study also has some limitations. First, since potential differences in practice settings (drug availability, analgesia protocols) that could influence outcomes, the single-centre design and modest sample size of the current study may limit generalizability, and control group with plain bupivacaine would have been valuable to evaluate the efficacy of the liposomal formulation. Further researches could be carried out to compare the LB and plain bupivacaine combined with epidural hydromorphone with multicentre or large cohort studies. Second, all participants fast from midnight according to the protocol of the study, however ERAS/ERAC recommendations clear fluids up to 2 h preoperatively. Third, the high cost of LB is a barrier to its widespread adoption, and further cost-effectiveness analyses are required. Fortunately, the price of liposomal bupivacaine in China has significantly dropped, and it now costs less than 54 USD. Finally, the duration of action of LB exceeds 72 h, a substantial proportion of women undergoing caesarean delivery are discharged prior to 72 h postoperatively. Consequently, we were unable to assess the analgesic efficacy of LB-enhance TAP block beyond 48 h. In future studies, postpartum pain situations can be followed up with telephone or online questionnaires for longer periods.

## 5 Conclusion

Epidural hydromorphone combined with LB-enhanced TAP block significantly prolongs analgesia duration and reduces opioid requirements during the first 24 h to 48 h after caesarean delivery. This regimen serves as a viable alternative for multimodal analgesia when ITM administration is precluded due to the unavailability of preservative-free morphine; however, its clinical implementation requires careful cost-benefit evaluation and patient preference assessment owing to the high cost of LB.

## Data Availability

The raw data supporting the conclusions of this article will be made available by the authors, without undue reservation.
